# Microfluidic Platforms for Exosome Engineering: Scalable Therapeutics for Cancer Immunotherapy and Infectious Diseases

**DOI:** 10.3390/ijms27146298

**Published:** 2026-07-15

**Authors:** Minyoung Lee, Kwangmin Park, Jungho Kim, Kyung-A Hyun, Anbazhagan Sathiyaseelan, Sunyoung Park

**Affiliations:** 1 Department of Biomedical Technology, Kangwon National University, Chuncheon 24341, Republic of Korea; anssow@naver.com; 2 Department of Clinical Laboratory Science, College of Health Sciences, Catholic University of Pusan, Busan 46252, Republic of Korea; pkmchi777@naver.com (K.P.);; 3 School of Biopharmaceutical and Medical Sciences, Sungshin Women’s University, 55 Dobong-ro 76 ga-gil, Gangbuk-gu, Seoul 01133, Republic of Korea

**Keywords:** exosomes, microfluidic engineering, cancer immunotherapy, infectious disease vaccines, clinical translation

## Abstract

Extracellular vesicles (EVs), particularly small EVs or exosomes, are promising cell-free therapeutics with superior biocompatibility and intrinsic targeting for synthetic nanoparticles. However, conventional bulk preparation methods suffer from low yield, poor reproducibility, and structural instability. Microfluidic technologies resolve these issues by enabling precise, automated, and low-shear fluidic manipulation. This mini-review highlights recent advances in microfluidic-engineered exosomes for cancer immunotherapy and infectious diseases. We evaluate critical microfluidic strategies for isolation, surface engineering, and cargo loading, contrasting platforms like ExoArc, acoustofluidics, cellular nanoporation, and electroporation. Particular emphasis is placed on complex modalities, including immune cell-derived exosomes (IEX), neo-antigen presentation, chimeric antigen receptor (CAR)-derived exosomes, and targeted siRNA delivery networks. Crucially, we analyze the technological disconnect between analytical microfluidic scales and massive therapeutic manufacturing volumes, addressing how physical forces risk damaging conformationally sensitive surface proteins (e.g., CAR scFv). Finally, we outline future perspectives, including high-throughput 3D-multiplexed networks, stimulus-responsive scarless elution, and integrated “sample-to-therapy” circuits. Guided by the MISEV2023 guidelines, this review frames the path toward standardized, clinical-scale engineering of multi-functional, cell-free immunotherapies.

## 1. Introduction

Exosomes are nanosized extracellular vesicles (EVs) ranging from approximately 30–150 nm (a lipid-bound particle) in diameter that originate from the endosomal pathway through the inward budding of multivesicular bodies (MVBs) [1,2,3]. Once considered merely cellular waste products, exosomes are now recognized as highly sophisticated mediators of intercellular communication capable of transferring diverse bioactive cargos, including proteins, lipids, DNA fragments, messenger RNA (mRNA), microRNA (miRNA), and small interfering RNA (siRNA) to recipient cells [4,5,6]. In addition, EVs have a set of surface marker proteins such as Alix, TSG101, HSC70, heat shock (Hsp90, Hsp70, and Hsp60), transmembrane tetraspanins (CD9, CD63, CD81 and CD82), CD37, and LAMP1 [7]. Owing to their intrinsic cell-derived membrane architecture, low immunogenicity, high biocompatibility, and natural ability to traverse biological barriers such as the blood–brain barrier (BBB), exosomes have emerged as a promising next-generation platform for drug delivery, immunomodulation, and cell-free therapeutics [8,9]. Compared with synthetic nanocarriers, exosomes exhibit superior physiological stability, prolonged circulation potential, and enhanced cellular uptake through membrane fusion and receptor-mediated internalization pathways. These unique biological properties have accelerated interest in engineered exosomes for applications in cancer immunotherapy, RNA delivery, regenerative medicine, and infectious disease vaccines [10,11,12,13]. In particular, exosome-based therapeutics provide a versatile platform for targeted delivery of nucleic acids and immunoregulatory biomolecules while minimizing systemic toxicity and immunogenic complications commonly associated with conventional nanoparticle systems [14].

Despite these advantages, the clinical translation of exosome therapeutics remains severely constrained by major manufacturing and engineering limitations. Conventional exosome isolation techniques, including ultracentrifugation (UC), density-gradient centrifugation, filtration, size-exclusion chromatography (SEC), precipitation, and immunoaffinity-based methods, often require prolonged processing times (>2 to 18 h), large sample volumes, and multiple purification steps while exposing vesicles to high shear stress (up to 100,000 g) and aggregation-related structural damage and often yield recovery rates below 10% with high lipoprotein contamination [15,16]. Furthermore, conventional cargo-loading approaches such as passive incubation and bulk electroporation frequently suffer from poor encapsulation efficiency, cargo instability, vesicle aggregation, and limited reproducibility [17]. Additional translational barriers include exosome heterogeneity (25–150 nm size range), batch-to-batch variability, rapid clearance by the mononuclear phagocyte system (MPS), and the lack of scalable Good Manufacturing Practice (GMP)-compatible production workflows. To address these shortcomings, microfluidic technologies have emerged as robust engineering platforms for precise and scalable exosome manipulation. Microfluidic systems enable highly controlled fluid dynamics at the microscale, allowing continuous-flow isolation, high-efficiency cargo loading, real-time process monitoring, and programmable surface engineering within integrated platforms [18]. Advanced microfluidic strategies, including acoustofluidics, deterministic lateral displacement (DLD), cellular nanoporation, and microfluidic electroporation, have significantly improved exosome yield, purity, loading efficiency, and functional reproducibility compared with conventional benchtop techniques [16,18,19,20,21]. Importantly, the integration of automation, multiplexing capability, and inline quality control positions microfluidics as a key enabling technology for the clinical-scale manufacturing of engineered exosome therapeutics.

In this mini-review, we examine recent advances in microfluidic-engineered exosome platforms with a particular focus on therapeutic applications in cancer immunotherapy and infectious diseases. We further highlight emerging strategies for exosome isolation, cargo loading, and surface functionalization while addressing current translational challenges related to large-scale manufacturing, biosafety evaluation, regulatory standardization, and clinical implementation.

## 2. Quantitative Advances in Microfluidic Exosome Engineering

### 2.1. Isolation of Exosomes

**Figure 1:**
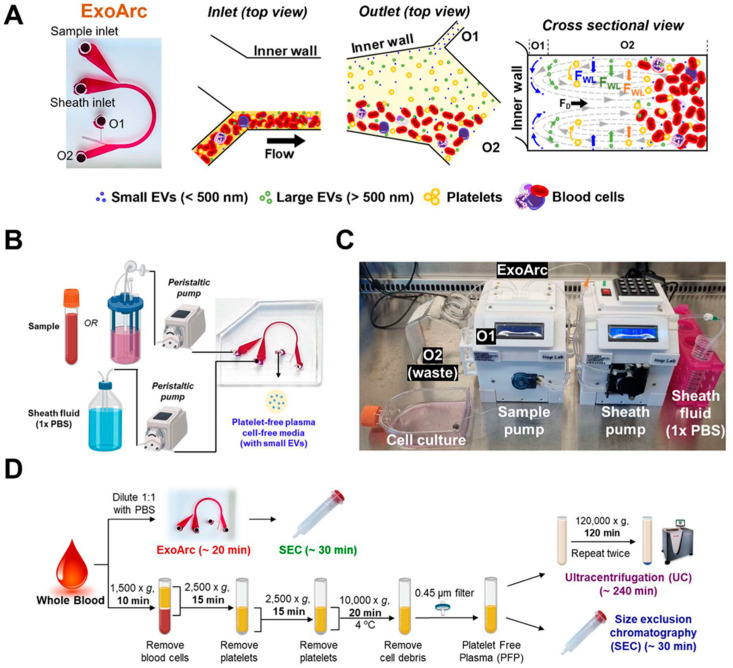
Microfluidic ExoArc platform for high-purity isolation of extracellular vesicles (EVs) from biological samples. (**A**) Schematic illustration of the ExoArc microfluidic device and its inertial microfluidics-based separation principle. Small extracellular vesicles (sEVs) are selectively enriched in the plasma fraction, while larger cellular components are removed through waste outlets. (**B**) Schematic representation of a portable closed-loop processing system integrated with the ExoArc platform for continuous sample handling and EV isolation under sterile conditions. (**C**) Photograph of the integrated microfluidic processing setup. (**D**) Comparative overview of EV isolation workflows employing ExoArc, size-exclusion chromatography (SEC), combined ExoArc–SEC processing, and conventional ultracentrifugation (UC) [9]. Adapted with permission from Ref. [9]. Copyright 2024 American Chemical Society. License Number 6280041081746.

The efficient isolation of high-purity EVs is a critical prerequisite for the successful development of exosome-based therapeutics. Among emerging microfluidic technologies, ExoArc and other label-free inertial microfluidic platforms have demonstrated exceptional separation performance (Table 1). The ExoArc platform enables rapid, automated, and high-purity EV isolation, highlighting the potential of microfluidic technologies for scalable and clinically translatable exosome manufacturing. The system exploits size-dependent hydrodynamic forces, including wall-induced lift forces and Dean flow-induced drag forces, to continuously separate blood cells, platelets, and extracellular vesicles (Figure 1). Operating at a high throughput of 0.1–0.2 mL/min, ExoArc achieves a submicrometer size cutoff of approximately 500 nm through Dean vortex-mediated particle focusing. Notably, the platform achieves complete removal of leukocytes and red blood cells while eliminating approximately 99.99% of platelets and platelet fragments [9]. Furthermore, when integrated with size-exclusion chromatography (SEC), the combined workflow enables EV isolation within approximately 50 min, yielding nearly 10-fold higher EV concentrations (∼3 × 10^7^ particles/mL of whole blood) compared with conventional ultracentrifugation (∼4.7 × 10^6^ particles/mL of whole blood), while maintaining minimal protein contamination (∼43 μg/mL) [9].

**Figure 2:**
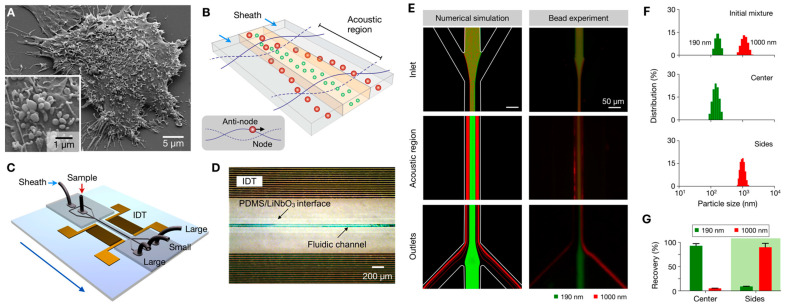
Acoustofluidic nanofilter platform for size-selective isolation of extracellular vesicles. (**A**) Representative scanning electron microscopy image showing extracellular microvesicles (MVs) released from human glioblastoma cells, highlighting their heterogeneous nanoscale dimensions. (**B**) Schematic illustration of the acoustofluidic separation mechanism. (**C**) Design of the acoustofluidic nanofilter incorporating interdigitated transducer (IDT) electrodes to generate standing surface acoustic waves across the microchannel. (**D**) Photograph of the integrated acoustofluidic device fabricated on a piezoelectric substrate and coupled with a bonded microfluidic channel. (**E**) Experimental and computational validation of size-dependent particle trajectories, demonstrating effective separation of nanoscale and microscale particles. (**F**) Particle size analysis confirming selective enrichment of vesicle populations following acoustofluidic filtration. (**G**) Evaluation of device performance showing high recovery efficiency and minimal sample loss during processing [21]. Adapted with permission from Ref. [21]. Copyright 2015 American Chemical Society. License Number 6280050661719.

Acoustofluidic platforms have emerged as powerful tools for extracellular vesicle isolation, engineering, and drug delivery applications. By utilizing acoustic radiation forces (ARF) and acoustic streaming at frequencies ranging from 1–10 MHz, these systems enable rapid, label-free separation and concentration of exosomes with recovery rates exceeding 80%, while preserving vesicle integrity and avoiding the extreme mechanical shear stresses (~100,000× *g*) associated with conventional ultracentrifugation (Table 1) [21,22]. Unlike conventional size-based filtration methods, acoustic separation offers tunable control through adjustment of acoustic field parameters, enabling dynamic regulation of separation selectivity [21,22]. Upon exposure to acoustic fields, vesicles undergo size-dependent migration toward pressure nodes, allowing continuous fractionation of extracellular vesicle subpopulations according to particle size. Larger vesicles experience stronger acoustic forces and are preferentially displaced toward sheath-flow regions, whereas smaller vesicles remain in the central flow stream [21]. Optimization of acoustic transducer design and energy transfer efficiency has enabled separation yields exceeding 90% while providing real-time control of vesicle size-selection thresholds [21]. Compared with ultracentrifugation and membrane filtration, acoustofluidic separation offers rapid processing, minimal vesicle damage, reduced sample requirements, and tunable size fractionation (Figure 2). Furthermore, the technology can be adapted for the selective isolation of multiple extracellular vesicle populations, including exosomes, microvesicles, and apoptotic bodies, and may be integrated with biosensing and molecular analysis modules for portable lab-on-a-chip applications [21]. Beyond isolation, acoustofluidic systems have also demonstrated significant potential for simultaneous drug loading and exosome encapsulation. By leveraging acoustic forces and microscale fluid dynamics, therapeutic agents, nanoparticles, and exosomes can be rapidly assembled into hybrid exosome-coated nanocarriers with enhanced loading efficiency [22]. These engineered nanocarriers exhibit improved cellular uptake, efficient intracellular drug delivery, and enhanced therapeutic efficacy compared with conventional loading approaches. The rapid, scalable, and chemical-free nature of acoustofluidic processing highlights its promise for the development of advanced exosome-based therapeutic and diagnostic platforms [22].

### 2.2. Cargo Loading Strategies

Loading therapeutic payloads, such as siRNA, mRNA, proteins, and small-molecule drugs, into exosomes remains challenging due to the structural integrity and limited permeability of the exosomal lipid bilayer. To address these limitations, microfluidic-based cargo loading strategies have emerged as powerful tools for improving loading efficiency while preserving vesicle integrity (Table 1). Among these, cellular nanoporation (CNP) employs microfluidic devices containing nanochannels (~500 nm diameter) that deliver short, high-intensity electrical pulses (200–400 V, 100–200 μs) directly to donor cells, inducing transient membrane permeabilization without compromising cell viability (Table 1). Unlike conventional bulk electroporation, CNP applies localized electric fields that enable controlled intracellular delivery of nucleic acids while maintaining cell viability above 90%, thereby supporting repeated stimulation and enhanced exosome production [23,24,25]. Consequently, CNP has been reported to increase exosome secretion by approximately 50-fold while achieving mRNA loading efficiencies of 2–10 intact mRNA copies per exosome, representing a 2000–10,000-fold improvement compared with naturally secreted vesicles. Furthermore, the loaded mRNA exhibited greater than 85% retention after 72 h of storage at 4 °C, demonstrating excellent cargo stability [25].

**Figure 3:**
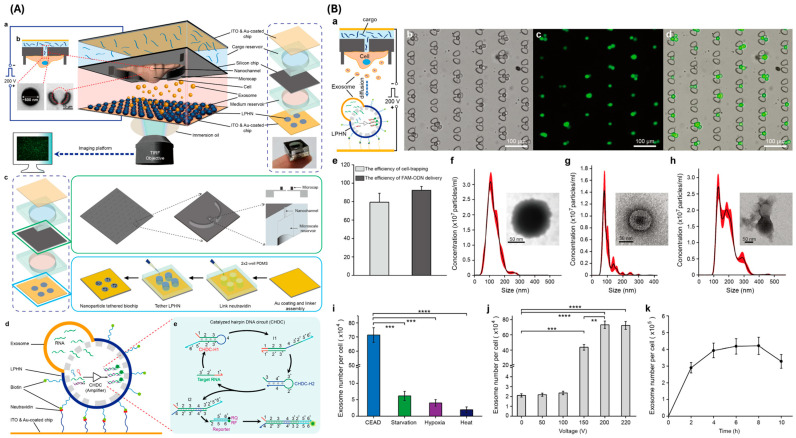
Cellular Nanoporation and Exosome Assessment Device (CEAD) for exosome engineering and real-time RNA analysis. (**A**) Principle and characterization of the CEAD platform. (**a**) Schematic illustration of the CEAD for cellular nanoporation and exosomal RNA analysis. (**b**) SEM image of the nanochannel/microcap array for single-cell trapping. (**c**) Fabrication of the nanochannel/microcap array and nanoparticle-tethered biochip. (**d**) Schematic of the catalytic hairpin DNA circuit (CHDC) for exosomal RNA signal amplification. (**e**) CHDC-mediated toehold strand-displacement reaction enabling amplified fluorescence detection of target exosomal RNA [19]. (**B**) Functional evaluation of the CEAD. (**a**) Schematic of nanochannel-mediated cargo transfection and exosome detection. (**b**–**d**) Intracellular delivery of fluorescence-labeled oligonucleotides. (**e**) Cell capture and cargo delivery efficiencies. (**f**–**h**) Characterization of lipid–polymer hybrid nanoparticles (LPHNs), exosomes, and LPHN–exosome complexes by nanoparticle tracking analysis and TEM. (**i**) Comparison of exosome secretion between CEAD and conventional stress-induced methods. (**j**) Voltage-dependent exosome production. (**k**) Time-dependent enhancement of exosome secretion following CEAD treatment. Data are presented as the mean ± SD of three technical replicates (*n* = 3). Statistical significance was determined using a two-tailed Student’s *t*-test (** *p* < 0.01, *** *p* < 0.001, **** *p* < 0.001) [19]. Adapted with permission from Ref. [19]. Copyright 2022 American Chemical Society. License Number 6280051486959.

A significant advancement of this technology is the Cellular Nanoporation and Exosome Assessment Device (CEAD), which integrates nanochannel electroporation with real-time exosomal RNA analysis within a single microfluidic platform [19]. CEAD demonstrated cell capture efficiencies of approximately 75–90% and transfection efficiencies approaching 90–100%, enabling precise and efficient intracellular cargo delivery (Figure 3). Importantly, the nanochannel-intensified electric field significantly enhanced exosome biogenesis, producing nearly 10-fold higher exosome yields than conventional stress-induced methods, including hypoxia, starvation, and heat treatment, while maintaining vesicle sizes within the characteristic exosomal range (70–110 nm). In addition, the platform incorporates a catalytic hairpin DNA circuit (CHDC)-based sensing system capable of sensitive and real-time monitoring of exosomal RNA expression following cellular engineering. The integration of efficient cargo loading, enhanced exosome production, and in situ molecular analysis highlights the potential of CEAD as a scalable and multifunctional platform for therapeutic exosome manufacturing and quality assessment [19]. 

**Figure 4:**
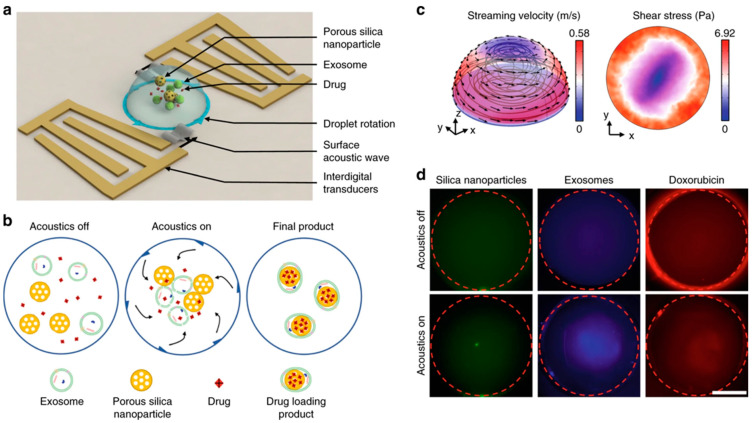
Acoustofluidic platform for simultaneous drug loading and exosome encapsulation. (**a**) Schematic illustration of an acoustofluidic device utilizing surface acoustic waves to induce droplet rotation and acoustic microstreaming, enabling the concentration and interaction of exosomes, therapeutic agents, and nanoparticle carriers. (**b**) Working mechanism of acoustofluidic-assisted drug loading and exosome coating to generate hybrid nanocarriers. (**c**) Numerical simulation of acoustic microstreaming and shear stress distribution within the droplet. (**d**) Representative fluorescence images showing enhanced concentration and co-localization of nanoparticles, exosomes, and drug molecules following acoustofluidic activation [22]. Reproduced from [22], licensed under CC BY 4.0.

Beyond electroporation-based approaches, acoustofluidic drug-loading systems have also been developed to simultaneously achieve drug loading and exosome encapsulation (Table 1). By leveraging acoustic radiation forces, acoustic microstreaming, and shear-induced mixing, therapeutic agents (e.g., doxorubicin), nanoparticles, and exosomes can be rapidly assembled into hybrid exosome-coated nanocarriers (Figure 4). This acoustofluidic strategy achieves drug-loading efficiencies of approximately 30% within 2 min, representing a substantial improvement over passive incubation methods, which typically achieve only ~0.08% loading efficiency after 22 h [22]. The resulting exosome-encapsulated nanocarriers exhibit enhanced cellular uptake, efficient intracellular drug delivery, and improved therapeutic efficacy, highlighting the promise of acoustofluidic technologies for advanced exosome-based drug delivery applications [22].

### 2.3. Surface Engineering and On-Demand Surface Engineering

**Figure 5:**
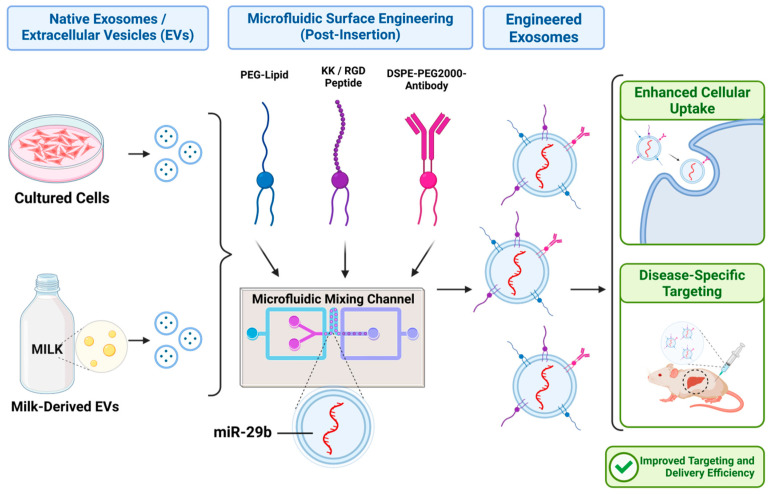
Microfluidic strategies for scalable exosome engineering and surface functionalization. The figure was created using BioRender (https://www.biorender.com/; accessed on 26 June 2026).

Surface functionalization of exosomes is essential for tissue-specific targeting, cellular uptake enhancement, immune evasion, and enabling their tracking (Table 1). This process is achieved through chemical (PEGylation, click chemistry, thiol-maleimide reaction, EDC/NHS chemistry, ligand-receptor reaction), physical methods (e.g., extrusion, ultrasound, and freeze–thaw cycles) and biological strategies (e.g., lentiviral transduction, plasmid transfection). These engineering approaches transform natural cell-to-cell communicators into personalized, precision-engineered therapeutic vehicles for oncology, immunology, and regenerative medicine [26,27,28]. Exosome surface engineering through microfluidic devices utilizes lab-on-a-chip technologies to precisely decorate extracellular vesicles with functional ligands, targeting peptides, and therapeutic payloads (Figure 5). This automated approach bypasses the low efficiency and heterogeneity issues of bulk methods like co-incubation, drastically improving targeted drug delivery and precision medicine [29,30]. Microfluidic on-demand exosome surface engineering represents the intersection of the two concepts: using miniaturized lab-on-a-chip technology to isolate, modify, and release customized therapeutic exosomes within a single, automated, and real-time workflow [31]. Traditional benchtop surface engineering methods (like ultracentrifugation, transfection, or chemical incubation) are heavily bottlenecked by low purity, long processing times, and potential damage to the vesicles. Microfluidics circumvents these limitations by controlling fluids at the micrometer scale, enabling highly precise, high-throughput molecular editing [32]. For example,

(i) Antigen Presentation and Immunogenic Targeting: Using 3D-printed microfluidic cell culture chips integrated with photo-cleavable linkers and immunomagnetic beads, the study conjugated tumor antigenic peptides (e.g., MAGE-A3, gp-100, and MART-1) to exosome surfaces [31]. This continuous-flow process: (1) captures exosomes via MHC-I-specific binding (~95% capture efficiency); (2) conjugates peptides through UV-light-triggered linker release (λ = 365 nm, <2 min); and (3) releases peptide-conjugated exosomes via controlled photocleavage with ~95% recovery. This integrated workflow reduces manual processing time from 4 to 6 h to real-time operation, improving yield and maintaining exosome integrity [31].

(ii) “Don’t Eat Me” Signals and Immune Evasion: Systemic administration of engineered exosomes faces rapid clearance by the mononuclear phagocyte system (MPS) [33,34]. Microfluidic surface engineering incorporating CD47 (a “don’t eat me” signal) extends in vivo circulatory half-life by 3-fold, allowing accumulation at target tissues [35]. Acoustic-driven surface modification can simultaneously conjugate CD47, targeting ligands, and immunogenic cargo within a single microfluidic device. A nanosecond pulsed microfluidic system (T-nsPMs) was developed for the high-throughput production of engineered exosomes with enhanced drug-loading efficiency. The engineered exosomes were co-functionalized with a 5HT1D antibody and CD47 protein to improve activated hepatic stellate cell targeting, extracellular matrix penetration, and immune evasion. miR-29b-loaded exosomes effectively suppressed hepatic stellate cell activation and reduced fibrotic markers, including α-SMA, COL1A1, TIMP-1, and p-SMAD2. Furthermore, the engineered exosomes significantly attenuated collagen deposition by inhibiting the TGF-β/SMAD signaling pathway, demonstrating strong antifibrotic potential [35].

(iii) Multivalent Targeting: Microfluidic approaches enable precise stoichiometric control of multiple surface ligands. For example, a microfluidic post-insertion strategy was developed for the simultaneous incorporation of polyethylene glycol lipids and KK- or RGD-modified high-functionality and quality (HFQ) lipids into milk-derived extracellular vesicles (EVs). In this approach, PEG and HFQ lipid micelles were rapidly mixed with EV suspensions using a microfluidic device, enabling efficient surface functionalization compared to conventional bulk mixing. The modified EVs demonstrated enhanced cellular association, tunable uptake behavior, and improved targeting capability while potentially reducing reticuloendothelial clearance. Furthermore, the microfluidic platform successfully incorporated poorly soluble RGD-modified lipids, highlighting its scalability and versatility for engineering functional EV-based drug delivery systems [36]. A universal microfluidic platform (ExoSE) was developed for multifunctional surface engineering of small extracellular vesicles (sEVs) through the integration of nanofluidic mechanoporation and microfluidic ligand conjugation. The ExoSE device enabled highly efficient incorporation of functionalized lipids into sEV membranes, achieving insertion efficiencies above 97% for both HEK293T- and milk-derived sEVs, significantly outperforming conventional co-incubation methods. Furthermore, the platform facilitated rapid covalent attachment of targeting ligands, including peptides (RGE) and aptamers (AS1411), resulting in enhanced cellular targeting and transmembrane transport specific to breast cancer cells (77.8%) compared to normal breast cells (32.5%). Engineered sEVs demonstrated improved glioma penetration, selective breast cancer targeting, and increased brain accumulation in vivo without detectable toxicity, highlighting the scalability and translational potential of the microfluidic engineering strategy for precision therapeutic delivery [30].

**Table 1:** Representative microfluidic strategies for exosome isolation, cargo loading, and surface engineering.

Microfluidic Strategy	Main Function	Key Advantages	Major Limitations	Therapeutic Relevance	Cost	Ref.
**Isolation Technologies**						
Size-based microfluidic isolation	Isolation of EVs using size-dependent separation	High purity, rapid processing, reduced shear damage	Channel clogging and limited large-scale validation	Preparation of clinical-grade exosomes	Low–Medium	[18,37]
**Cargo Loading Technologies**						
Acoustofluidic loading	Simultaneous cargo loading and vesicle manipulation using acoustic forces	Rapid loading with improved vesicle integrity	Requires optimization of acoustic parameters	Drug and nucleic acid delivery	High	[18,22]
Cellular nanoporation	Stimulates source cells to produce cargo-enriched exosomes	Extremely high mRNA loading efficiency and enhanced exosome secretion	Product heterogeneity and scalability challenges	mRNA therapeutics and cancer immunotherapy	Very high	[25,38]
Microfluidic electroporation	Electrical permeabilization for therapeutic cargo encapsulation	Improved loading efficiency compared to bulk electroporation	Potential membrane instability and cargo aggregation	siRNA and RNA-based therapies	High–Very high	[38,39]
**Surface Engineering Technologies**						
On-demand surface engineering	Controlled exosome surface functionalization with ligands or antigens	Precise targeting and immune modulation	Mostly proof-of-concept stage	Targeted cancer immunotherapy and vaccines	Medium–High	[30,31]

## 3. Microfluidic-Engineered Exosomes in Cancer Immunotherapy

### 3.1. Immune Cell-Derived Exosomes (IEX) and Neoantigen Presentation

Immune cell-derived exosomes (IEX), particularly dendritic cell-derived exosomes (DEX), inherently express MHC-I/II and co-stimulatory molecules (CD80, CD86), making them potent cell-free vaccines [40,41,42]. Microfluidic platforms efficiently pulse DCs with tumor lysates on-chip, rapidly harvesting highly immunogenic DEX that directly cross-present antigens to activate cytotoxic CD8+ T cells [31]. Because exosomes lack live-cell machinery, they bypass the severe immunosuppressive networks of the tumor microenvironment (TME), maintaining their anti-tumor efficacy [14].

### 3.2. Chimeric Antigen Receptor (CAR) Exosomes

**Figure 6:**
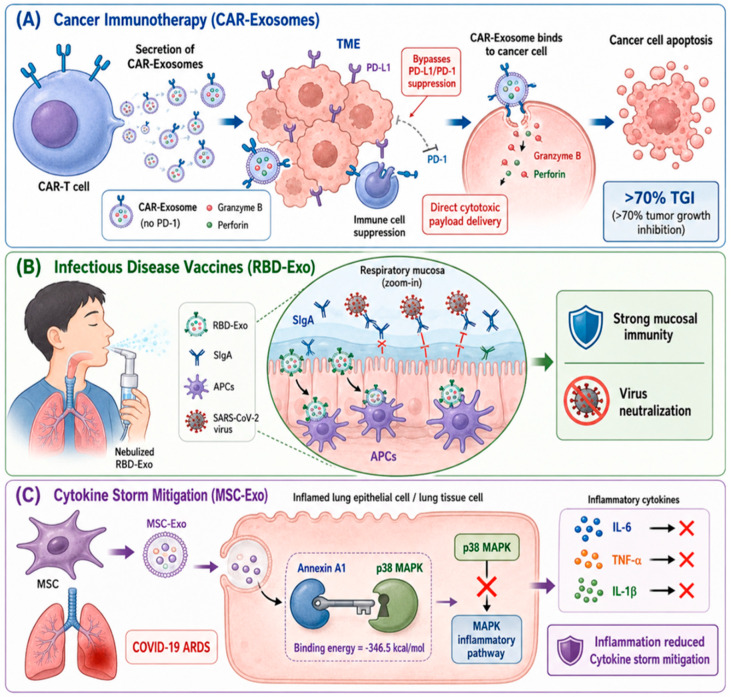
Therapeutic mechanisms of microfluidic-engineered exosomes in cancer and infectious diseases. This illustration was prepared by the authors with assistance from ChatGPT (OpenAI, GPT-5.5 Thinking, medium reasoning level, https://openai.com/ accessed on 1 June 2026).

CAR T-cell therapy is a highly personalized form of immunotherapy that genetically modifies a patient’s own T-cells in a lab to supercharge their ability to find and destroy cancer cells. It has revolutionized the treatment of relapsed or refractory blood cancers, such as certain leukemias, lymphomas, and multiple myeloma [42]. However, the common risks include cytokine release syndrome (CRS), a systemic inflammatory response causing high fevers and low blood pressure, and neurological toxicities. Because the treatment often eradicates healthy immune cells alongside cancer cells, patients remain immunosuppressed and vulnerable to infections for months after [43]. Alternatively, CAR-T cell-derived exosomes are a cutting-edge, cell-free alternative to traditional CAR-T cell therapy (Figure 6). Furthermore, CAR-exosome efficacy correlates with CAR surface density; optimal loading achieves 15–25 CAR molecules per exosome. The absence of inhibitory receptors (PD-1, TIGIT) on exosome surfaces, combined with high perforin/granzyme B content, enables serial tumor cell killing without the autoimmune complications of parental CAR-T cells, such as CRS or neurotoxicity [44,45]. Similarly, a recent study demonstrated that CAR-derived exosomes express high levels of cytotoxic molecules that contribute to tumor suppression while lacking PD-1 expression, unlike their parental CAR-T cells [46]. Furthermore, the study evidenced that these CAR exosomes carry approximately 0.69 ng of CAR protein per µg of exosome, determined by ELISA. Preclinical in vivo models using CAR-T cell exosomes in cancer therapy show that administering 100–150 µg of these CAR exosomes with different transmembrane domains yields approximately 67% to 70% tumor growth inhibition (TGI) in solid tumors (such as human EGFR+ and HER2+ xenografts). In toxicity studies, mice receiving the maximum tolerated dose (MTD; 10 or 45 million CAR-T cells) died within 48 h due to cytokine release syndrome (CRS), whereas mice treated with 150 μg of CAR-derived exosomes exhibited negligible mortality, less than 20% body weight loss, and reduced secretion of inflammatory cytokines, including IFN-γ and IL-2 [46]. However, the isolation of CAR exosomes and purification has been highly complex and is transforming into clinical applications [44]. Furthermore, according to the literature search, no direct studies were conducted on the isolation of CAR cells-derived exosomes using microfluidic techniques.

### 3.3. siRNA Delivery

Small interfering RNAs (siRNAs) have immense potential to silence “undruggable” cancer genes (e.g., mutant KRAS, c-Myc) [17]. Exosome-mediated siRNA delivery overcomes the instability and immunogenicity of synthetic lipid nanoparticles. Several studies have utilized the electroporation method for the preparation of siRNA-loaded exosomes [47,48]. The study evidenced that engineered exosomes loaded via electroporation have successfully delivered KRAS-targeting siRNA to pancreatic cancer cells, showing superior target knockdown compared to liposomes [34]. In addition, acoustic shock waves are utilized for the production of siRNA-loaded exosomes. For example, a scalable shock wave extracellular vesicle engineering technology (SWEET) was developed to efficiently encapsulate therapeutic siRNA into small bovine milk-derived extracellular vesicles (sBMEVs) using acoustic shock waves. This non-genetic platform achieved high-efficiency loading of KRASG12C-targeting siRNA while maintaining EV integrity and enabling large-scale production. The engineered sBMEVs effectively silenced oncogenic KRASG12C expression in cancer cells and, following intravenous administration, significantly suppressed tumor growth in a non-small cell lung cancer xenograft mouse model [49]. However, a major limitation of bulk electroporation, acoustic shock wave loading, and related techniques is the tendency of siRNA to aggregate during the loading process. Additionally, the actual siRNA encapsulation efficiency is frequently not quantified or clearly reported. Microfluidic platforms offer a potential solution by enabling precise control over fluid dynamics and cargo loading, thereby reducing aggregation, improving loading uniformity, and enhancing the therapeutic availability of siRNA at target tissues. For example, an ultrasonic microfluidic platform was developed to enhance the loading of siHSP47 into human embryonic kidney cell-derived exosomes (293F-EXOs), achieving a loading efficiency of 31.1%. The engineered EXO-siHSP47 effectively penetrated collagen barriers and silenced HSP47 expression in TGF-β1-activated fibroblasts. Consequently, the treatment significantly reduced extracellular matrix protein secretion and deposition while inhibiting fibroblast differentiation and migration. These findings highlight the potential of microfluidic-assisted exosome engineering as an efficient strategy for siRNA delivery in idiopathic pulmonary fibrosis therapy [50].

Beyond siRNA encapsulation, integrated microfluidic systems have also been developed for the efficient preparation of drug-loaded exosomes. One platform combined exosome drug loading, magnetic separation, and electrochemical quantification within a single device, achieving enhanced doxorubicin loading through a 3D chaotic flow mixer and enabling up to 90% killing of doxorubicin-resistant breast cancer cells when combined with magnetic hyperthermia [51]. Another integrated microfluidic chip enabled simultaneous exosome isolation and drug loading by coupling immunomagnetic separation with microelectroporation, achieving a separation purity of 82.74% and a drug encapsulation efficiency of 14.01% [52]. These studies highlight the potential of microfluidic integration to streamline exosome manufacturing, improve loading efficiency, and facilitate clinical translation of exosome-based therapeutics.

## 4. Microfluidic-Engineered Exosomes for Infectious Diseases

Beyond oncology, microfluidic-engineered exosomes are increasingly explored for infectious disease applications, particularly in mucosal vaccination and host-directed immunomodulation. Although the following therapeutic examples primarily highlight the biological efficacy of engineered exosomes, they are discussed here because recent advances in microfluidic isolation, cargo loading, and surface engineering have substantially improved their manufacturing efficiency, reproducibility, and translational potential. Accordingly, the focus of this section is to illustrate how microfluidic technologies facilitate the development of next-generation exosome therapeutics [18,23,32].

### 4.1. Inhalable Exosome-Based Viral Vaccines

The COVID-19 pandemic exposed key limitations of lipid nanoparticle (LNP)-based mRNA vaccines, including cold-chain dependence and limited induction of mucosal immunity. To address these challenges, researchers developed an inhalable virus-like particle (VLP) vaccine by conjugating the SARS-CoV-2 receptor-binding domain (RBD) onto lung-derived exosomes (RBD-Exo) [53]. The optimized formulation achieved a conjugation efficiency of 10.5%, corresponding to approximately 892 RBD molecules per exosome. Notably, the lyophilized RBD-Exo preparations maintained antigen integrity and structural stability at 21 °C (room temperature) for >3 months without significant loss of RBD immunogenicity, a marked advantage over cold-chain-dependent LNP platforms. Intranasal delivery via nebulization elicited significantly enhanced mucosal immunity, with 10–100-fold higher secretory IgA (SIgA) titers in respiratory secretions at 2 weeks post-boost, compared to intramuscular injection. In live SARS-CoV-2 challenge hamster models (*n* = 6 per group), RBD-Exo immunization achieved a 3.43-fold reduction in nasal and lung viral RNA copies by day 7, complete clearance of infectious pseudovirus, and marked attenuation of severe pneumonia (histological score reduced by 70%), with no vaccine-associated enhanced respiratory disease (VAERD) [53]. In parallel with these biological advances, microfluidic engineering enables precise control over vesicle size, antigen loading, and formulation reproducibility, facilitating scalable manufacturing and quality assurance of inhalable exosome vaccines for future clinical translation [30,32,53].

### 4.2. Cytokine Storm Mitigation

In severe infectious diseases like COVID-19 or sepsis, mortality is often driven by a hyperinflammatory “cytokine storm.” Mesenchymal stem cell-derived exosomes (MSC-Exos) demonstrate profound immunomodulatory effects [54]. MSC-Exos demonstrated potent immunomodulatory effects against SARS-CoV-2-induced cytokine storm through inhibition of the MAPK signaling pathway [55]. Molecular docking analyses revealed strong interactions between exosomal proteins (Annexin A1 and TGF-β) and key MAPK components, including p38, ERK1/2, and JNK1, suggesting direct pathway modulation. In SARS-CoV-2-infected hamster models, MSC-Exo treatment significantly downregulated MAPK-related genes (MEKK1, MEKK2, and MEKK3), reduced phosphorylation of JNK1, p38, and ERK1/2, and suppressed the production of pro-inflammatory cytokines such as IL-1β, IL-6, and TNF-α. These molecular changes were accompanied by improved lung histopathology, including reduced alveolar wall thickening and decreased inflammatory cell infiltration, highlighting the therapeutic potential of MSC-Exos for mitigating severe COVID-19-associated ARDS [55]. Recent progress in continuous-flow microfluidic manufacturing and automated process control is expected to improve the reproducibility, scalability, and quality consistency of MSC-derived exosome therapeutics, thereby supporting future GMP-compatible clinical translation [18,32]. The recent advances and representative studies discussed in this section are summarized in Table 2, highlighting the diverse therapeutic applications and outcomes achieved using microfluidic-engineered exosomes.

## 5. Current Challenges and Clinical Translation

### 5.1. Manufacturing and Scalability Challenges

Despite the remarkable precision and reproducibility offered by microfluidic technologies, large-scale manufacturing remains a major barrier to the clinical translation of engineered exosome therapeutics. Therapeutic administration typically requires doses ranging from 10^12^ to 10^15^ particles per patient, varying by route of administration and indication, necessitating the processing of substantial source material volumes [70]. Although microfluidic platforms provide superior control over isolation, cargo loading, and surface engineering, most current systems remain limited in throughput when operated as single-channel devices; for reference, a single ExoArc channel operating at 0.1–0.2 mL/min would require 8–167 h to process source volumes sufficient for a single clinical dose, underscoring the need for parallelization.

To address this, significant efforts have focused on parallelized and continuous-flow manufacturing strategies. Multiplexed microfluidic architectures operating hundreds of channels simultaneously have demonstrated the capacity to scale production while maintaining process consistency. Similarly, the integration of microfluidic flow-control principles with hollow-fiber bioreactor systems enables continuous exosome production under defined culture conditions. Microfluidic extrusion of living cells can generate approximately 100–250-fold higher particle yields than naturally secreted exosomes while preserving key biological properties, including surface marker expression (CD63, CD81, TSG101) and cellular uptake efficiency [71]. Additionally, lyophilization has demonstrated promise for maintaining exosomal structural integrity at room temperature (>3 months for vaccine-type preparations); however, systematic validation of cryoprotectant formulations (e.g., sucrose, trehalose) across diverse therapeutic exosome subtypes remains necessary.

Beyond production yield, exosome heterogeneity remains a significant translational challenge. Variations in donor cell sources, culture conditions, isolation protocols, and cargo-loading strategies can result in substantial batch-to-batch variability in exosome composition and therapeutic efficacy, complicating quality control and regulatory approval. Although automated continuous-flow microfluidic systems offer improved process consistency, standardized platforms, particularly for immune cell-derived exosome production, remain limited. Manufacturing challenges will be essential for achieving reproducible, scalable manufacturing of clinical-grade exosome therapeutics.

### 5.2. Biological Performance, Biodistribution, and Biosafety

A comprehensive understanding of exosome pharmacokinetics, biodistribution, and long-term safety is essential for successful clinical implementation. Following systemic administration, native exosomes exhibit short circulation half-lives of 5–30 min and are rapidly sequestered by the mononuclear phagocyte system (MPS), resulting in predominant hepatic and splenic accumulation; consequently, only a limited fraction reaches the intended target tissue. Compounding this, intravenously administered exosomes rapidly adsorb serum proteins, forming a dynamic protein corona of albumin, immunoglobulins, and complement factors, which can mask engineered surface ligands, promote opsonization, and redirect biodistribution unpredictably [72,73]. Microfluidic surface-engineering strategies have emerged as powerful tools for enhancing in vivo performance. Functionalization with immune-evasive molecules such as CD47 has been shown to reduce macrophage-mediated clearance, prolonging circulatory half-life by up to 3-fold and improving target tissue accumulation [23]. Precise conjugation of targeting ligands further enhances tissue specificity; blood–brain barrier (BBB)-targeting peptides, including rabies virus glycoprotein (RVG) and apolipoprotein E3 (apoE3)-derived sequences, which engage LRP1-mediated transcytosis on brain endothelial cells, have shown promise for improving central nervous system delivery [74]. Nevertheless, precise control over ligand density, orientation, and receptor-binding activity remains necessary to achieve predictable biodistribution and therapeutic outcomes.

Beyond biodistribution, biosafety is a critical consideration. Exosomal nucleic acids (ssRNA, CpG DNA) can activate endosomal Toll-like receptors (TLR3, TLR7/8, TLR9) and cytosolic sensors (STING, RIG-I), triggering interferon and inflammatory responses; while exploitable in cancer vaccine contexts, such innate immune activation requires suppression in anti-inflammatory applications such as MSC-exosome therapy [75,76]. Comprehensive immunogenicity, toxicity, and repeated-dose safety assessments are therefore required before clinical implementation. Current in vivo tracking approaches include near-infrared (NIR) fluorescence imaging (DiR, Cy5), radiolabeling for PET (^89^Zr-oxine) and SPECT (^111^In-oxine), and bioluminescence-based imaging [77,78]; however, standardized pharmacokinetic assessment methodologies aligned with regulatory requirements remain to be established.

### 5.3. Regulatory Standardization and GMP Compliance

The successful clinical translation of engineered exosomes requires rigorous standardization, quality control, and regulatory compliance. The MISEV2023 guidelines provide a comprehensive characterization framework, recommending systematic evaluation of identity, purity, potency, safety, and stability [71]. Identity assessment requires both positive markers, transmembrane proteins (CD9, CD63, CD81), and cytosolic markers (TSG101, HSP70), as well as negative markers confirming the absence of contaminating organelles, including calnexin (endoplasmic reticulum), apolipoprotein B (lipoprotein particles), and histone H3 (apoptotic bodies). Purity assessment focuses on minimizing contamination from lipoproteins, protein aggregates, and residual cellular components, while potency assays should be directly linked to the therapeutic mechanism of action. Critical quality attributes (CQAs), including particle size distribution, concentration, morphology, aggregation state, cargo stability, surface phenotype, and targeting-ligand functionality, directly influence therapeutic potency, biodistribution, safety, and product reproducibility. Microfluidic technologies offer distinct advantages for GMP-compatible production: automated processing, closed-system manufacturing, and integrated Process Analytical Technology (PAT) tools enable real-time CQA monitoring, supporting batch-to-batch consistency and regulatory compliance. Commercially, platforms such as ExCoBio’s ExoOnAbsolute system and Evox Therapeutics’ GMP-grade exosome manufacturing workflows have demonstrated scalable clinical-grade production [79,80].

Regulatory classification varies by jurisdiction: in the United States, engineered exosomes are generally regulated as biological products requiring Biologics License Application (BLA) review under 21 CFR 601, while in Europe they qualify as Advanced Therapy Medicinal Products (ATMPs) under EMA Regulation EC 1394/2007. Alignment with ICH Q8–Q11 pharmaceutical quality guidelines alongside MISEV2023 standards will be essential for global regulatory harmonization [81]. Although several exosome-based platforms have advanced to early clinical evaluation (Table 3), widespread adoption remains constrained by manufacturing scalability, product heterogeneity, dose standardization, and long-term safety [82]. Addressing these challenges through integrated microfluidic manufacturing, validated quality systems, and harmonized regulatory frameworks will be essential for accelerating the clinical translation of engineered exosome therapeutics.

## 6. Future Perspectives

To bridge the current translational gap, next-generation microfluidic architectures must transition from rigid analytical devices into highly scalable, biocompatible manufacturing units (Table 4). Overcoming the volumetric throughput bottleneck requires the development of massively parallelized microfluidic networks often referred to as 3D-multiplexed or “numbered-up” chips capable of processing therapeutic-scale fluid volumes (liters per hour) without compromising the precise fluid dynamics of single channels. To address the molecular degradation caused by harsh chemical isolation or aggressive loading forces, the integration of stimulus-responsive biomaterials offers a highly promising pathway. Utilizing photo-cleavable or enzymatically degradable linkers on affinity-capture surfaces will allow for the gentle, scarless release of high-purity CAR exosomes, leaving their delicate surface single-chain variable fragments (scFv) and internal cytotoxic payloads entirely intact. Similarly, for cargo-loading, the field is shifting toward non-destructive membrane-permeabilization strategies. Novel droplet-based microfluidics and localized nano-electroporation arrays can apply highly confined, ultra-short electrical pulses that transiently open targeted pores in the lipid bilayer. This localized energy delivery maximizes the encapsulation efficiency of large macromolecules (such as CRISPR-Cas9 complexes or mRNA) while minimizing the global thermal and mechanical stress that would otherwise denature conformationally sensitive CAR proteins. Finally, the ultimate evolution of this field lies in the realization of fully closed, integrated “Sample-to-Therapy” platforms. By placing high-throughput physical sorting, gentle affinity-refinement, automated cargo-loading, and continuous on-chip quality control (QC) profiling into a single, uninterrupted fluidic circuit, researchers can establish a standardized, automated pipeline. This consolidated approach will dramatically minimize product loss, eliminate the human error inherent in multi-step manual handling, and establish microfluidics as a viable foundation for clinical-scale, cell-free CAR immunotherapy manufacturing.

## 7. Conclusions

Microfluidic technology has fundamentally shifted the paradigm of exosome research from basic analytical isolation to the quantitative, on-demand engineering of sophisticated cell-free therapeutics. By enabling high-throughput isolation (e.g., ExoArc), exponentially increasing cargo loading efficiency (e.g., CNP, Acoustofluidics), and facilitating precise surface modification, microfluidics addresses the critical limitations of conventional methods. As demonstrated by the >70% tumor growth inhibition in CAR-exosome models and the 100-fold increase in mucosal immunity via inhalable viral vaccines, engineered exosomes possess immense clinical power. Moving forward, resolving the engineering challenges of scaling up to particles/dose and adhering to GMP/MISEV2023 standards will be paramount. Ultimately, microfluidic-engineered exosomes hold the transformative potential to become the next generation of precision medicine.

## Figures and Tables

**Table 2:** Representative therapeutic applications of microfluidic-engineered exosomes in cancer immunotherapy and infectious diseases.

Applications	Exosome Source	Engineering Strategy	Therapeutic Cargo	Key Outcome (In Vitro/In Vivo)	Clinical Phase	Ref.
Cancer Immunotherapy						
Breast cancer metastasis	Engineered DC-derived exosomes	CCR7/PD-1 surface engineering	STING agonist	LN remodeling and suppression of tumor progression and metastasis	Preclinical	[56]
Immune checkpoint therapy	Gene-engineered exosomes	PD-1 gene engineering + immune adjuvant encapsulation	PD-1 + imiquimod	Reversal of T-cell exhaustion and enhanced antitumor immunity	Preclinical	[57]
NSCLC/solid tumors	Genetically engineered NK-derived exosomes	NK-exosome genetic engineering	NKG2D + IL-24	Enhanced tumor targeting, increased apoptosis, and improved antitumor potency	Preclinical	[58]
Personalized cancer vaccines	Tumor-derived engineered EVs	Scaffold vaccine engineering	Tumor-derived antigens	Robust and durable antitumor immunity	Preclinical	[59]
Infectious Disease						
SARS-CoV-2 T-cell vaccine model	SARS-CoV-2 Spike-carrying EVs	Antigen loading/display on EVs	SARS-CoV-2 Spike antigen	T-cell activation in human PBMC-based immunogenicity model	Preclinical/in vitro	[60]
Pulmonary COVID-19 vaccine	Genetically engineered dendritic cell-derived exosomes	Fc-mediated surface display and pulmonary delivery enhancement	Fc-Lamp2b-RBD fusion protein	Enhanced epithelial-layer transmission and lung distribution	Preclinical/in vivo	[61]
Multivalent respiratory virus vaccine	Exosome-based protein vaccine platform	Surface display of multiple viral antigens	SARS-CoV-2, influenza, and RSV antigens	Induction of humoral and cellular immune responses against multiple respiratory viral antigens	Preclinical/in vivo	[62]
SARS-CoV-2 exosomal T-cell epitope vaccine	Exosomal vaccine platform	T-cell epitope loading combined with antibody-inducing vaccination	SARS-CoV-2 T-cell epitopes and antibody-inducing antigens	Synergistic immune protection in highly humanized mice	Preclinical/in vivo	[63]
SARS-CoV-2 and influenza-associated inflammation	Mesenchymal stem cell-derived EVs	Immunomodulatory EV treatment	miR-146a/NF-κB pathway modulation	Reduced inflammatory responses to SARS-CoV-2 and influenza viral proteins	Preclinical/in vitro	[64]
Cytokine storm/acute lung injury	Inflammatory cytokine-primed MSC-derived EVs	Cytokine priming to enhance EV immunomodulation	Anti-inflammatory miRNA-enriched EV cargo	Reduced inflammatory cytokines, immune-cell recruitment, pulmonary edema, and viral protein-induced inflammatory damage	Preclinical/in vivo	[65]
Polymicrobial bacterial sepsis	Probiotic bacteria-released EVs	pH-conditioned probiotic BEV treatment	FPR1/2 pathway activation	Enhanced macrophage phagocytosis, bacterial clearance, survival, and reduced inflammatory injury	Preclinical/in vivo	[66]
Bacterial sepsis	Engineered EVs	Antimicrobial peptide coating	Cationic antimicrobial peptide-coated EVs	Improved antibacterial activity while preserving cytoprotective and anti-inflammatory effects	Preclinical/in vitro	[67]
Bacterial wound infection	Edwardsiella piscicida-derived EVs	Antimicrobial peptide loading	Cathelicidin LL37-loaded bacterial EVs	Promoted antibacterial and wound-healing activity	Preclinical/in vitro	[68]
Viral infection and antiviral EV application	Therapeutic or virus-associated EVs	Antiviral EV-based delivery or decoy strategy	Antiviral cargo/viral-entry interference targets	EVs summarized as antiviral tools and therapeutic targets for viral infection management	Review/translational strategy	[69]

**Table 3:** Key manufacturing, engineering, and regulatory challenges associated with microfluidic-engineered exosomes and corresponding translational strategies [79,80,83].

Translational Challenge	Underlying Cause	Impact on Therapy	Microfluidic Solution	Remaining Limitation
Low exosome yield	Limited natural exosome secretion and inefficient isolation	Insufficient therapeutic dose production	Continuous-flow microfluidics and cellular nanoporation	Large-scale manufacturing remains challenging
Cargo loading inefficiency	Poor membrane permeability and cargo leakage	Reduced therapeutic potency	Microfluidic electroporation and acoustofluidic loading	Potential membrane destabilization and variable encapsulation
Exosome heterogeneity	Variability in source cells and isolation methods	Inconsistent therapeutic efficacy	Size-selective and controlled microfluidic separation	Standardized quality control is still lacking
Structural damage during processing	High shear stress and harsh centrifugation conditions	Loss of vesicle integrity and bioactivity	Low-shear microfluidic manipulation platforms	Long-term stability requires further validation
Rapid in vivo clearance	Uptake by the mononuclear phagocyte system (MPS)	Reduced circulation time and target accumulation	Surface engineering with targeting or “don’t eat me” ligands	Biodistribution control remains incomplete
Batch-to-batch variability	Manual processing and inconsistent operating conditions	Poor reproducibility and regulatory concerns	Automated continuous-flow microfluidic systems	GMP-scale standardization is still limited
Risk of contamination	Protein impurities, lipoproteins, and residual free cargo	Safety concerns and misleading potency evaluation	Integrated purification and inline monitoring systems	Sterility assurance and validation remain necessary
Clinical translation and GMP compliance	Lack of standardized manufacturing protocols	Delayed regulatory approval and commercialization	Process-integrated microfluidic manufacturing and PAT tools	Regulatory frameworks for EV therapeutics are still evolving

**Table 4:** Representative clinical and preclinical exosome therapeutic platforms and their translational challenges [84,85].

Product Type	Company/Trial	Indication	Engineering Strategy	Clinical Phase	Major Challenge
MSC-derived exosomes	ExoFlo™/ Direct Biologics	COVID-19 and ARDS	Native immunomodulatory EV therapy	Clinical evaluation	Product heterogeneity and potency standardization
Dendritic cell-derived exosomes	DexVac platform	Cancer immunotherapy	Antigen-presenting exosome vaccines	Early clinical studies	Limited therapeutic efficacy and scalable production
Plant-derived exosome therapeutics	Various exploratory platforms	Oral inflammatory diseases and drug delivery	Natural vesicle-mediated cargo delivery	Preclinical/experimental	Standardized isolation and characterization
Engineered CAR-exosomes	Academic/preclinical platforms	Solid tumors and hematological malignancies	Surface CAR expression and cytotoxic cargo delivery	Advanced preclinical	Target specificity and large-scale manufacturing
Exosome-based RNA delivery systems	Codiak Biosciences	Oncology and genetics disorders	Engineered RNA-loaded exosomes	Early clinical/preclinical	Cargo loading reproducibility and regulatory approval
Inhalable exosome vaccines	RBD-Exo platforms	COVID-19 and respiratory infections	Antigen-decorated inhalable exosomes	Advanced preclinical	Dose standardization and long-term biosafety

## Data Availability

No new data were created or analyzed in this study. Data sharing is not applicable to this article.

## Abbreviations

The following abbreviations are used in this manuscript:

CAR	Chimeric Antigen Receptor
DLD	Deterministic Lateral Displacement
EVs	Extracellular Vesicles
GMP	Good Manufacturing Practice
IEX	Immune Cell-Derived Exosomes
MHC	Major Histocompatibility Complex
MISEV	Minimal Information for Studies of Extracellular Vesicles
NK	Natural Killer (as in NK cells)
scFv	Single-Chain Variable Fragment
siRNA	Small Interfering RNA
TFF	Tangential Flow Filtration
QC	Quality Control
